# [1,2-Bis(1*H*-benzimidazol-2-yl-κ*N* ^3^)ethane]dichloridozinc(II)

**DOI:** 10.1107/S1600536809052738

**Published:** 2009-12-12

**Authors:** Yan-Ling Zhou, Ming-Hua Zeng, Seik Weng Ng

**Affiliations:** a School of Chemistry & Chemical Engineering, Guangxi Normal University, 541004 Guilin 541004, People’s Republic of China; b Department of Chemistry, University of Malaya, 50603 Kuala Lumpur, Malaysia

## Abstract

The title compound, [ZnCl_2_(C_16_H_14_N_4_)], crystallizes with two mol­ecules in the asymmetric unit. The Zn^II^ atoms show distorted tetrahedral coordination environments. Adjacent mol­ecules are linked by N—H⋯Cl hydrogen bonds, forming a three-dimensional network.

## Related literature

For the synthesis of the ligand, see: van Albada *et al.* (1995 ▶). For the zinc dichloride adduct of a similar *N*-heterocycle, see: Zhou *et al.* (2010 ▶).
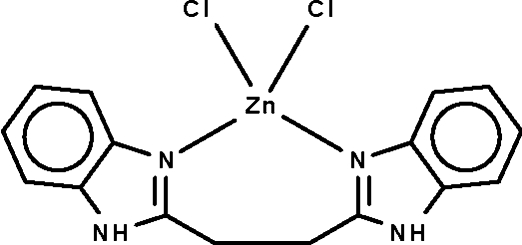


## Experimental

### 

#### Crystal data

[ZnCl_2_(C_16_H_14_N_4_)]
*M* *_r_* = 398.58
Monoclinic, 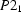

*a* = 8.0868 (4) Å
*b* = 13.8605 (8) Å
*c* = 14.8504 (8) Å
β = 92.664 (1)°
*V* = 1662.7 (2) Å^3^
*Z* = 4
Mo *K*α radiation
μ = 1.80 mm^−1^
*T* = 293 K
0.48 × 0.34 × 0.30 mm

#### Data collection

Bruker SMART diffractometer
Absorption correction: multi-scan (*SADABS*; Sheldrick, 1996 ▶) *T*_min_ = 0.479, *T*_max_ = 0.614
8555 measured reflections
5820 independent reflections
4956 reflections with *I* > 2σ(*I*)
*R*_int_ = 0.018

#### Refinement

*R*[*F* ^2^ > 2σ(*F* ^2^)] = 0.029
*wR*(*F* ^2^) = 0.077
*S* = 0.97
5820 reflections
415 parameters
1 restraint
H-atom parameters constrained
Δρ_max_ = 0.30 e Å^−3^
Δρ_min_ = −0.30 e Å^−3^
Absolute structure: Flack (1983 ▶), 2049 Friedel pairs
Flack parameter: 0.1 (1)

### 

Data collection: *SMART* (Bruker, 2001 ▶); cell refinement: *SAINT* (Bruker, 2001 ▶); data reduction: *SAINT*; program(s) used to solve structure: *SHELXS97* (Sheldrick, 2008 ▶); program(s) used to refine structure: *SHELXL97* (Sheldrick, 2008 ▶); molecular graphics: *X-SEED* (Barbour, 2001 ▶); software used to prepare material for publication: *publCIF* (Westrip, 2009 ▶).

## Supplementary Material

Crystal structure: contains datablocks I, global. DOI: 10.1107/S1600536809052738/bt5133sup1.cif

Structure factors: contains datablocks I. DOI: 10.1107/S1600536809052738/bt5133Isup2.hkl

Additional supplementary materials:  crystallographic information; 3D view; checkCIF report

## Figures and Tables

**Table 1:** Hydrogen-bond geometry (Å, °)

*D*—H⋯*A*	*D*—H	H⋯*A*	*D*⋯*A*	*D*—H⋯*A*
N2—H2⋯Cl3	0.86	2.64	3.316 (3)	137
N3—H3⋯Cl4^i^	0.86	2.76	3.291 (3)	121
N6—H6⋯Cl1^ii^	0.86	2.39	3.225 (3)	164
N7—H7⋯Cl2^iii^	0.86	2.52	3.277 (3)	148

Symmetry codes: (i) 
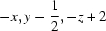
; (ii) 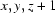; (iii) 
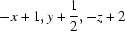
.

**Fig. 1.**
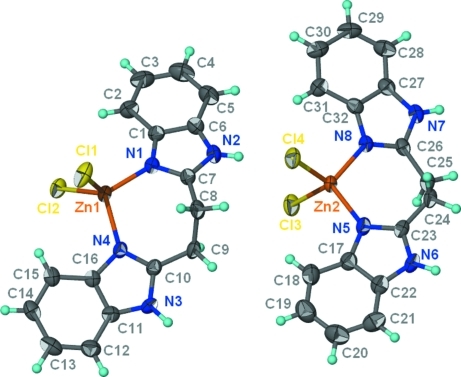
Anisotropic displacement ellipsoid plot (Barbour, 2001) of the two independent molecules of ZnCl2(C16H14N4) at the 50% probability level; hydrogen atoms are drawn as sphere of arbitrary radius.

## supplementary crystallographic information

### Experimental

1,4-Bis(2-benzimidazolyl)ethanebutane was synthesized by using a literature method (van Albada *et al.*, 1995). To a solution of zinc chloride hexahydrate (0.25 g, 1 mmol) in ethanol (3 ml) was added an aqueous solution (4 ml) of the ligand (0.24 g, 1 mmol). The reactants were sealed in a 15-ml Teflon-lined, stainless-steel Parr bomb. The bomb was heated at 413 K for 3 days. The cool solution yielded single crystals in *ca* 30% yield.

### Refinement

Hydrogen atoms were generated geometrically and were constrained to ride on their parent atoms [C–H = 0.95–0.99 Å; N–H 0.88 Å *U*_iso_(H) = 1.2*U*_eq_(C, N)].

### Crystal data

**Table:**

[ZnCl_2_(C_16_H_14_N_4_)]	*F*(000) = 808
*M**_r_* = 398.58	*D*_x_ = 1.592 Mg m^−^^3^
Monoclinic, *P*2_1_	Mo *K*α radiation, λ = 0.71073 Å
Hall symbol: P 2yb	Cell parameters from 4769 reflections
*a* = 8.0868 (4) Å	θ = 2.5–27.1°
*b* = 13.8605 (8) Å	µ = 1.80 mm^−^^1^
*c* = 14.8504 (8) Å	*T* = 293 K
β = 92.664 (1)°	Block, colorless
*V* = 1662.7 (2) Å^3^	0.48 × 0.34 × 0.30 mm
*Z* = 4	

### Data collection

**Table:**

Bruker SMART diffractometer	5820 independent reflections
Radiation source: fine-focus sealed tube	4956 reflections with *I* > 2σ(*I*)
graphite	*R*_int_ = 0.018
φ and ω scans	θ_max_ = 27.1°, θ_min_ = 1.4°
Absorption correction: multi-scan (*SADABS*; Sheldrick, 1996)	*h* = −10→10
*T*_min_ = 0.479, *T*_max_ = 0.614	*k* = −17→13
8555 measured reflections	*l* = −18→15

### Refinement

**Table:**

Refinement on *F*^2^	Secondary atom site location: difference Fourier map
Least-squares matrix: full	Hydrogen site location: inferred from neighbouring sites
*R*[*F*^2^ > 2σ(*F*^2^)] = 0.029	H-atom parameters constrained
*wR*(*F*^2^) = 0.077	*w* = 1/[σ^2^(*F*_o_^2^) + (0.0406*P*)^2^] where *P* = (*F*_o_^2^ + 2*F*_c_^2^)/3
*S* = 0.97	(Δ/σ)_max_ = 0.002
5820 reflections	Δρ_max_ = 0.30 e Å^−^^3^
415 parameters	Δρ_min_ = −0.30 e Å^−^^3^
1 restraint	Absolute structure: Flack (1983), 2049 Friedel pairs
Primary atom site location: structure-invariant direct methods	Flack parameter: 0.1 (1)

### Fractional atomic coordinates and isotropic or equivalent isotropic displacement parameters (Å^2^)

**Table:**

	*x*	*y*	*z*	*U*_iso_*/*U*_eq_	
Zn1	0.24147 (5)	0.50006 (3)	0.64876 (2)	0.03664 (11)	
Zn2	0.19950 (5)	0.65365 (3)	1.15403 (2)	0.03913 (11)	
Cl1	−0.01667 (11)	0.50457 (12)	0.58667 (6)	0.0633 (3)	
Cl2	0.43158 (10)	0.50750 (9)	0.54292 (5)	0.0475 (2)	
Cl3	0.42290 (13)	0.60363 (9)	1.08123 (6)	0.0558 (3)	
Cl4	−0.01188 (12)	0.67049 (9)	1.05101 (6)	0.0528 (3)	
N1	0.2783 (4)	0.6025 (2)	0.74082 (19)	0.0409 (7)	
N2	0.2933 (4)	0.6691 (3)	0.8750 (2)	0.0508 (8)	
H2	0.2833	0.6764	0.9320	0.061*	
N3	0.2869 (4)	0.2549 (2)	0.8128 (2)	0.0447 (8)	
H3	0.2932	0.2255	0.8638	0.054*	
N4	0.2675 (4)	0.3733 (2)	0.71535 (18)	0.0362 (7)	
N5	0.1409 (4)	0.5614 (3)	1.25109 (18)	0.0425 (8)	
N6	0.0915 (4)	0.4941 (3)	1.38061 (18)	0.0450 (7)	
H6	0.0781	0.4883	1.4375	0.054*	
N7	0.3232 (4)	0.8927 (3)	1.3116 (2)	0.0423 (8)	
H7	0.3505	0.9194	1.3623	0.051*	
N8	0.2503 (4)	0.7799 (2)	1.21321 (19)	0.0393 (7)	
C1	0.3522 (5)	0.6924 (3)	0.7341 (2)	0.0412 (9)	
C2	0.4079 (5)	0.7424 (3)	0.6600 (3)	0.0556 (11)	
H2A	0.4011	0.7154	0.6026	0.067*	
C3	0.4724 (6)	0.8320 (4)	0.6743 (3)	0.0649 (13)	
H3A	0.5124	0.8660	0.6259	0.078*	
C4	0.4803 (6)	0.8741 (4)	0.7593 (4)	0.0697 (13)	
H4	0.5237	0.9359	0.7662	0.084*	
C5	0.4259 (6)	0.8269 (4)	0.8327 (3)	0.0627 (12)	
H5	0.4321	0.8550	0.8897	0.075*	
C6	0.3612 (5)	0.7356 (3)	0.8188 (3)	0.0465 (10)	
C7	0.2453 (4)	0.5917 (3)	0.8278 (2)	0.0393 (8)	
C8	0.1625 (5)	0.5068 (3)	0.8669 (2)	0.0474 (9)	
H8A	0.0683	0.4893	0.8275	0.057*	
H8B	0.1204	0.5254	0.9245	0.057*	
C9	0.2725 (5)	0.4180 (3)	0.8812 (2)	0.0467 (10)	
H9A	0.3847	0.4394	0.8959	0.056*	
H9B	0.2350	0.3824	0.9327	0.056*	
C10	0.2755 (4)	0.3506 (3)	0.8020 (2)	0.0379 (8)	
C11	0.2870 (5)	0.2110 (3)	0.7301 (3)	0.0419 (9)	
C12	0.2914 (5)	0.1150 (3)	0.7039 (3)	0.0526 (11)	
H12	0.2971	0.0652	0.7459	0.063*	
C13	0.2871 (5)	0.0971 (3)	0.6128 (3)	0.0572 (12)	
H13	0.2904	0.0336	0.5927	0.069*	
C14	0.2778 (5)	0.1719 (3)	0.5497 (3)	0.0503 (11)	
H14	0.2759	0.1569	0.4886	0.060*	
C15	0.2714 (5)	0.2670 (3)	0.5756 (3)	0.0460 (10)	
H15	0.2648	0.3165	0.5333	0.055*	
C16	0.2752 (4)	0.2864 (3)	0.6687 (2)	0.0367 (8)	
C17	0.1120 (5)	0.4632 (3)	1.2365 (3)	0.0449 (9)	
C18	0.1112 (6)	0.4077 (4)	1.1586 (3)	0.0608 (12)	
H18	0.1288	0.4354	1.1028	0.073*	
C19	0.0837 (6)	0.3114 (4)	1.1666 (4)	0.0710 (14)	
H19	0.0832	0.2729	1.1153	0.085*	
C20	0.0565 (6)	0.2690 (4)	1.2494 (3)	0.0703 (14)	
H20	0.0409	0.2026	1.2522	0.084*	
C21	0.0520 (5)	0.3218 (4)	1.3262 (3)	0.0625 (12)	
H21	0.0306	0.2933	1.3812	0.075*	
C22	0.0806 (5)	0.4199 (3)	1.3191 (3)	0.0464 (10)	
C23	0.1259 (4)	0.5765 (3)	1.3389 (2)	0.0383 (8)	
C24	0.1397 (4)	0.6706 (3)	1.3870 (2)	0.0433 (9)	
H24A	0.1299	0.6588	1.4509	0.052*	
H24B	0.0469	0.7107	1.3669	0.052*	
C25	0.3010 (5)	0.7277 (3)	1.3742 (2)	0.0432 (9)	
H25A	0.3304	0.7617	1.4298	0.052*	
H25B	0.3892	0.6824	1.3634	0.052*	
C26	0.2912 (4)	0.7987 (3)	1.2992 (2)	0.0382 (8)	
C27	0.3052 (4)	0.9398 (3)	1.2296 (3)	0.0420 (9)	
C28	0.3225 (5)	1.0351 (3)	1.2046 (3)	0.0553 (11)	
H28	0.3498	1.0827	1.2467	0.066*	
C29	0.2976 (5)	1.0563 (3)	1.1143 (3)	0.0596 (12)	
H29	0.3090	1.1197	1.0949	0.071*	
C30	0.2561 (5)	0.9853 (4)	1.0518 (3)	0.0579 (12)	
H30	0.2421	1.0023	0.9914	0.069*	
C31	0.2346 (5)	0.8899 (3)	1.0759 (3)	0.0486 (10)	
H31	0.2055	0.8429	1.0334	0.058*	
C32	0.2591 (4)	0.8677 (3)	1.1681 (2)	0.0399 (9)	

### Atomic displacement parameters (Å^2^)

**Table:**

	*U*^11^	*U*^22^	*U*^33^	*U*^12^	*U*^13^	*U*^23^
Zn1	0.0431 (2)	0.0407 (2)	0.02621 (18)	0.0000 (2)	0.00288 (14)	0.00228 (18)
Zn2	0.0505 (2)	0.0409 (3)	0.02615 (18)	0.0009 (2)	0.00373 (15)	−0.00242 (18)
Cl1	0.0401 (5)	0.1098 (10)	0.0401 (5)	0.0026 (6)	0.0018 (4)	0.0161 (6)
Cl2	0.0464 (5)	0.0616 (6)	0.0353 (4)	−0.0007 (5)	0.0111 (3)	0.0066 (5)
Cl3	0.0548 (6)	0.0724 (8)	0.0410 (5)	0.0098 (5)	0.0106 (4)	−0.0068 (5)
Cl4	0.0539 (5)	0.0680 (8)	0.0358 (4)	0.0051 (5)	−0.0055 (4)	−0.0060 (5)
N1	0.0497 (18)	0.0401 (19)	0.0331 (15)	0.0015 (15)	0.0033 (13)	0.0006 (14)
N2	0.072 (2)	0.045 (2)	0.0350 (15)	−0.0021 (18)	0.0042 (14)	−0.0078 (15)
N3	0.058 (2)	0.040 (2)	0.0361 (17)	−0.0006 (16)	0.0062 (14)	0.0112 (15)
N4	0.0413 (16)	0.0388 (19)	0.0288 (15)	−0.0049 (14)	0.0043 (12)	0.0018 (13)
N5	0.0490 (18)	0.049 (2)	0.0300 (15)	−0.0036 (16)	0.0049 (13)	0.0002 (14)
N6	0.0483 (16)	0.058 (2)	0.0292 (14)	−0.0059 (18)	0.0031 (12)	0.0082 (16)
N7	0.0390 (16)	0.049 (2)	0.0384 (17)	0.0026 (15)	0.0006 (13)	−0.0096 (15)
N8	0.0461 (17)	0.039 (2)	0.0321 (16)	−0.0003 (14)	−0.0001 (13)	−0.0041 (14)
C1	0.044 (2)	0.039 (2)	0.0410 (19)	0.0053 (17)	0.0052 (16)	0.0032 (17)
C2	0.067 (3)	0.048 (3)	0.052 (2)	0.000 (2)	0.009 (2)	0.007 (2)
C3	0.063 (3)	0.051 (3)	0.082 (3)	0.002 (2)	0.015 (2)	0.020 (3)
C4	0.060 (3)	0.047 (3)	0.102 (4)	−0.005 (2)	0.000 (3)	−0.002 (3)
C5	0.074 (3)	0.047 (3)	0.067 (3)	−0.005 (2)	0.002 (2)	−0.011 (2)
C6	0.051 (2)	0.043 (2)	0.045 (2)	0.0038 (19)	0.0014 (18)	−0.0025 (18)
C7	0.042 (2)	0.041 (2)	0.0351 (18)	0.0051 (17)	0.0059 (15)	−0.0006 (16)
C8	0.057 (2)	0.051 (2)	0.0359 (17)	0.000 (2)	0.0175 (15)	−0.007 (2)
C9	0.065 (3)	0.050 (3)	0.0254 (18)	−0.007 (2)	0.0020 (16)	0.0026 (18)
C10	0.0391 (19)	0.041 (2)	0.0342 (19)	−0.0046 (17)	0.0063 (14)	0.0036 (17)
C11	0.038 (2)	0.040 (2)	0.047 (2)	0.0009 (17)	0.0056 (16)	0.0019 (18)
C12	0.056 (2)	0.041 (3)	0.061 (3)	−0.002 (2)	0.011 (2)	0.001 (2)
C13	0.048 (2)	0.043 (3)	0.082 (3)	−0.002 (2)	0.010 (2)	−0.014 (2)
C14	0.044 (2)	0.055 (3)	0.052 (2)	0.001 (2)	0.0078 (17)	−0.017 (2)
C15	0.044 (2)	0.054 (3)	0.040 (2)	−0.0032 (19)	0.0024 (17)	−0.0066 (19)
C16	0.0302 (17)	0.042 (2)	0.0384 (19)	−0.0021 (16)	0.0029 (14)	−0.0009 (17)
C17	0.045 (2)	0.047 (2)	0.042 (2)	−0.0060 (18)	−0.0013 (16)	−0.0003 (17)
C18	0.074 (3)	0.062 (3)	0.046 (2)	−0.007 (3)	0.002 (2)	−0.006 (2)
C19	0.078 (3)	0.063 (4)	0.072 (3)	−0.016 (3)	0.005 (3)	−0.019 (3)
C20	0.067 (3)	0.052 (3)	0.091 (4)	−0.011 (2)	−0.002 (3)	−0.007 (3)
C21	0.056 (3)	0.062 (3)	0.069 (3)	−0.010 (2)	0.000 (2)	0.017 (3)
C22	0.039 (2)	0.054 (3)	0.046 (2)	−0.0055 (19)	−0.0032 (16)	0.000 (2)
C23	0.0337 (18)	0.051 (2)	0.0306 (18)	0.0035 (17)	0.0015 (14)	0.0039 (16)
C24	0.0468 (19)	0.057 (3)	0.0269 (16)	0.0026 (19)	0.0077 (14)	−0.0013 (17)
C25	0.045 (2)	0.053 (3)	0.0311 (18)	−0.0007 (18)	−0.0024 (15)	−0.0045 (18)
C26	0.0343 (18)	0.043 (2)	0.038 (2)	0.0007 (16)	0.0065 (15)	−0.0086 (17)
C27	0.035 (2)	0.043 (3)	0.048 (2)	0.0037 (17)	0.0050 (16)	−0.0049 (19)
C28	0.048 (2)	0.039 (3)	0.078 (3)	−0.0045 (19)	−0.004 (2)	−0.006 (2)
C29	0.054 (3)	0.039 (3)	0.085 (3)	0.001 (2)	0.008 (2)	0.014 (3)
C30	0.057 (2)	0.053 (3)	0.064 (3)	0.007 (2)	0.003 (2)	0.017 (2)
C31	0.050 (2)	0.051 (3)	0.044 (2)	−0.001 (2)	0.0029 (17)	0.0048 (19)
C32	0.0368 (19)	0.037 (2)	0.046 (2)	0.0034 (17)	0.0072 (16)	−0.0038 (18)

### Geometric parameters (Å, °)

**Table:**

Zn1—N1	1.984 (3)	C8—H8B	0.9700
Zn1—N4	2.023 (3)	C9—C10	1.503 (5)
Zn1—Cl1	2.243 (1)	C9—H9A	0.9700
Zn1—Cl2	2.252 (1)	C9—H9B	0.9700
Zn2—N5	2.000 (3)	C11—C12	1.386 (6)
Zn2—N8	1.992 (3)	C11—C16	1.387 (5)
Zn2—Cl3	2.257 (1)	C12—C13	1.374 (6)
Zn2—Cl4	2.253 (1)	C12—H12	0.9300
N1—C7	1.339 (4)	C13—C14	1.397 (6)
N1—C1	1.388 (5)	C13—H13	0.9300
N2—C7	1.330 (5)	C14—C15	1.375 (6)
N2—C6	1.375 (5)	C14—H14	0.9300
N2—H2	0.8600	C15—C16	1.409 (5)
N3—C10	1.339 (5)	C15—H15	0.9300
N3—C11	1.370 (5)	C17—C18	1.390 (6)
N3—H3	0.8600	C17—C22	1.399 (6)
N4—C10	1.323 (4)	C18—C19	1.358 (7)
N4—C16	1.392 (5)	C18—H18	0.9300
N5—C23	1.332 (4)	C19—C20	1.390 (7)
N5—C17	1.397 (5)	C19—H19	0.9300
N6—C23	1.334 (5)	C20—C21	1.357 (7)
N6—C22	1.375 (5)	C20—H20	0.9300
N6—H6	0.8600	C21—C22	1.385 (6)
N7—C26	1.338 (5)	C21—H21	0.9300
N7—C27	1.384 (5)	C23—C24	1.489 (6)
N7—H7	0.8600	C24—C25	1.545 (5)
N8—C26	1.331 (4)	C24—H24A	0.9700
N8—C32	1.392 (5)	C24—H24B	0.9700
C1—C6	1.392 (5)	C25—C26	1.486 (6)
C1—C2	1.393 (5)	C25—H25A	0.9700
C2—C3	1.359 (7)	C25—H25B	0.9700
C2—H2A	0.9300	C27—C28	1.380 (6)
C3—C4	1.390 (7)	C27—C32	1.393 (5)
C3—H3A	0.9300	C28—C29	1.378 (6)
C4—C5	1.361 (7)	C28—H28	0.9300
C4—H4	0.9300	C29—C30	1.383 (7)
C5—C6	1.382 (6)	C29—H29	0.9300
C5—H5	0.9300	C30—C31	1.382 (6)
C7—C8	1.485 (6)	C30—H30	0.9300
C8—C9	1.527 (6)	C31—C32	1.408 (5)
C8—H8A	0.9700	C31—H31	0.9300
			
N1—Zn1—N4	106.03 (12)	N3—C11—C12	132.8 (4)
N1—Zn1—Cl1	111.74 (10)	N3—C11—C16	104.5 (3)
N4—Zn1—Cl1	107.38 (9)	C12—C11—C16	122.7 (4)
N1—Zn1—Cl2	111.27 (10)	C13—C12—C11	116.7 (4)
N4—Zn1—Cl2	108.80 (9)	C13—C12—H12	121.6
Cl1—Zn1—Cl2	111.37 (3)	C11—C12—H12	121.6
N8—Zn2—N5	107.11 (12)	C12—C13—C14	121.6 (4)
N8—Zn2—Cl4	110.05 (9)	C12—C13—H13	119.2
N5—Zn2—Cl4	110.98 (10)	C14—C13—H13	119.2
N8—Zn2—Cl3	109.15 (9)	C15—C14—C13	121.7 (4)
N5—Zn2—Cl3	111.76 (10)	C15—C14—H14	119.1
Cl4—Zn2—Cl3	107.79 (4)	C13—C14—H14	119.1
C7—N1—C1	106.0 (3)	C14—C15—C16	117.2 (4)
C7—N1—Zn1	123.7 (3)	C14—C15—H15	121.4
C1—N1—Zn1	130.1 (2)	C16—C15—H15	121.4
C7—N2—C6	109.6 (3)	C11—C16—N4	109.2 (3)
C7—N2—H2	125.2	C11—C16—C15	120.0 (4)
C6—N2—H2	125.2	N4—C16—C15	130.8 (4)
C10—N3—C11	109.7 (3)	C18—C17—N5	131.6 (4)
C10—N3—H3	125.2	C18—C17—C22	119.9 (4)
C11—N3—H3	125.2	N5—C17—C22	108.5 (4)
C10—N4—C16	106.1 (3)	C19—C18—C17	117.7 (5)
C10—N4—Zn1	132.9 (3)	C19—C18—H18	121.1
C16—N4—Zn1	121.0 (2)	C17—C18—H18	121.1
C23—N5—C17	106.4 (3)	C18—C19—C20	121.8 (5)
C23—N5—Zn2	129.9 (3)	C18—C19—H19	119.1
C17—N5—Zn2	123.7 (3)	C20—C19—H19	119.1
C23—N6—C22	109.9 (3)	C21—C20—C19	121.9 (5)
C23—N6—H6	125.1	C21—C20—H20	119.1
C22—N6—H6	125.1	C19—C20—H20	119.1
C26—N7—C27	109.1 (3)	C20—C21—C22	117.0 (5)
C26—N7—H7	125.5	C20—C21—H21	121.5
C27—N7—H7	125.5	C22—C21—H21	121.5
C26—N8—C32	105.9 (3)	N6—C22—C21	133.6 (4)
C26—N8—Zn2	129.3 (3)	N6—C22—C17	104.7 (4)
C32—N8—Zn2	124.7 (2)	C21—C22—C17	121.7 (4)
N1—C1—C6	109.0 (3)	N5—C23—N6	110.5 (4)
N1—C1—C2	131.2 (4)	N5—C23—C24	126.8 (4)
C6—C1—C2	119.7 (4)	N6—C23—C24	122.7 (3)
C3—C2—C1	117.8 (4)	C23—C24—C25	115.8 (3)
C3—C2—H2A	121.1	C23—C24—H24A	108.3
C1—C2—H2A	121.1	C25—C24—H24A	108.3
C2—C3—C4	121.8 (4)	C23—C24—H24B	108.3
C2—C3—H3A	119.1	C25—C24—H24B	108.3
C4—C3—H3A	119.1	H24A—C24—H24B	107.4
C5—C4—C3	121.6 (5)	C26—C25—C24	114.6 (3)
C5—C4—H4	119.2	C26—C25—H25A	108.6
C3—C4—H4	119.2	C24—C25—H25A	108.6
C4—C5—C6	117.0 (4)	C26—C25—H25B	108.6
C4—C5—H5	121.5	C24—C25—H25B	108.6
C6—C5—H5	121.5	H25A—C25—H25B	107.6
N2—C6—C5	133.0 (4)	N8—C26—N7	111.1 (4)
N2—C6—C1	104.8 (4)	N8—C26—C25	126.3 (4)
C5—C6—C1	122.2 (4)	N7—C26—C25	122.6 (3)
N2—C7—N1	110.6 (4)	C28—C27—N7	132.9 (4)
N2—C7—C8	123.9 (3)	C28—C27—C32	122.5 (4)
N1—C7—C8	125.4 (3)	N7—C27—C32	104.6 (4)
C7—C8—C9	115.0 (3)	C29—C28—C27	117.0 (4)
C7—C8—H8A	108.5	C29—C28—H28	121.5
C9—C8—H8A	108.5	C27—C28—H28	121.5
C7—C8—H8B	108.5	C28—C29—C30	121.3 (4)
C9—C8—H8B	108.5	C28—C29—H29	119.3
H8A—C8—H8B	107.5	C30—C29—H29	119.3
C10—C9—C8	115.0 (3)	C31—C30—C29	122.5 (4)
C10—C9—H9A	108.5	C31—C30—H30	118.8
C8—C9—H9A	108.5	C29—C30—H30	118.8
C10—C9—H9B	108.5	C30—C31—C32	116.6 (4)
C8—C9—H9B	108.5	C30—C31—H31	121.7
H9A—C9—H9B	107.5	C32—C31—H31	121.7
N4—C10—N3	110.6 (3)	N8—C32—C27	109.2 (3)
N4—C10—C9	127.7 (4)	N8—C32—C31	130.6 (4)
N3—C10—C9	121.7 (3)	C27—C32—C31	120.1 (4)
			
N4—Zn1—N1—C7	−28.8 (3)	C12—C13—C14—C15	−0.5 (6)
Cl1—Zn1—N1—C7	87.8 (3)	C13—C14—C15—C16	0.2 (6)
Cl2—Zn1—N1—C7	−147.0 (3)	N3—C11—C16—N4	−0.3 (4)
N4—Zn1—N1—C1	145.0 (3)	C12—C11—C16—N4	177.9 (4)
Cl1—Zn1—N1—C1	−98.3 (3)	N3—C11—C16—C15	−179.8 (3)
Cl2—Zn1—N1—C1	26.9 (3)	C12—C11—C16—C15	−1.6 (6)
N1—Zn1—N4—C10	12.5 (3)	C10—N4—C16—C11	0.4 (4)
Cl1—Zn1—N4—C10	−107.1 (3)	Zn1—N4—C16—C11	−177.9 (2)
Cl2—Zn1—N4—C10	132.2 (3)	C10—N4—C16—C15	179.8 (4)
N1—Zn1—N4—C16	−169.8 (3)	Zn1—N4—C16—C15	1.5 (5)
Cl1—Zn1—N4—C16	70.6 (3)	C14—C15—C16—C11	0.7 (5)
Cl2—Zn1—N4—C16	−50.0 (3)	C14—C15—C16—N4	−178.6 (3)
N8—Zn2—N5—C23	3.8 (4)	C23—N5—C17—C18	179.8 (4)
Cl4—Zn2—N5—C23	−116.4 (3)	Zn2—N5—C17—C18	−1.8 (6)
Cl3—Zn2—N5—C23	123.3 (3)	C23—N5—C17—C22	−0.6 (4)
N8—Zn2—N5—C17	−174.1 (3)	Zn2—N5—C17—C22	177.8 (2)
Cl4—Zn2—N5—C17	65.7 (3)	N5—C17—C18—C19	177.8 (5)
Cl3—Zn2—N5—C17	−54.6 (3)	C22—C17—C18—C19	−1.8 (7)
N5—Zn2—N8—C26	19.8 (3)	C17—C18—C19—C20	0.3 (8)
Cl4—Zn2—N8—C26	140.6 (3)	C18—C19—C20—C21	1.6 (8)
Cl3—Zn2—N8—C26	−101.3 (3)	C19—C20—C21—C22	−1.8 (7)
N5—Zn2—N8—C32	−164.5 (3)	C23—N6—C22—C21	178.4 (4)
Cl4—Zn2—N8—C32	−43.7 (3)	C23—N6—C22—C17	0.3 (4)
Cl3—Zn2—N8—C32	74.4 (3)	C20—C21—C22—N6	−177.4 (4)
C7—N1—C1—C6	−0.6 (4)	C20—C21—C22—C17	0.3 (6)
Zn1—N1—C1—C6	−175.3 (3)	C18—C17—C22—N6	179.8 (4)
C7—N1—C1—C2	−178.0 (4)	N5—C17—C22—N6	0.1 (4)
Zn1—N1—C1—C2	7.3 (6)	C18—C17—C22—C21	1.5 (6)
N1—C1—C2—C3	178.4 (4)	N5—C17—C22—C21	−178.2 (4)
C6—C1—C2—C3	1.2 (6)	C17—N5—C23—N6	0.8 (4)
C1—C2—C3—C4	−1.3 (7)	Zn2—N5—C23—N6	−177.4 (3)
C2—C3—C4—C5	1.0 (8)	C17—N5—C23—C24	−177.7 (4)
C3—C4—C5—C6	−0.5 (7)	Zn2—N5—C23—C24	4.1 (5)
C7—N2—C6—C5	178.6 (5)	C22—N6—C23—N5	−0.7 (4)
C7—N2—C6—C1	−0.5 (4)	C22—N6—C23—C24	177.9 (3)
C4—C5—C6—N2	−178.5 (5)	N5—C23—C24—C25	−54.7 (5)
C4—C5—C6—C1	0.4 (7)	N6—C23—C24—C25	127.0 (4)
N1—C1—C6—N2	0.7 (4)	C23—C24—C25—C26	92.8 (4)
C2—C1—C6—N2	178.4 (4)	C32—N8—C26—N7	0.8 (4)
N1—C1—C6—C5	−178.6 (4)	Zn2—N8—C26—N7	177.1 (2)
C2—C1—C6—C5	−0.8 (6)	C32—N8—C26—C25	−179.5 (3)
C6—N2—C7—N1	0.2 (5)	Zn2—N8—C26—C25	−3.2 (5)
C6—N2—C7—C8	−178.3 (4)	C27—N7—C26—N8	−0.8 (4)
C1—N1—C7—N2	0.2 (4)	C27—N7—C26—C25	179.5 (3)
Zn1—N1—C7—N2	175.4 (2)	C24—C25—C26—N8	−56.7 (5)
C1—N1—C7—C8	178.7 (4)	C24—C25—C26—N7	123.0 (4)
Zn1—N1—C7—C8	−6.2 (5)	C26—N7—C27—C28	179.8 (4)
N2—C7—C8—C9	−104.4 (4)	C26—N7—C27—C32	0.5 (4)
N1—C7—C8—C9	77.3 (5)	N7—C27—C28—C29	178.7 (4)
C7—C8—C9—C10	−88.8 (4)	C32—C27—C28—C29	−2.1 (6)
C16—N4—C10—N3	−0.3 (4)	C27—C28—C29—C30	0.4 (6)
Zn1—N4—C10—N3	177.6 (2)	C28—C29—C30—C31	1.0 (7)
C16—N4—C10—C9	179.9 (4)	C29—C30—C31—C32	−0.7 (6)
Zn1—N4—C10—C9	−2.2 (6)	C26—N8—C32—C27	−0.5 (4)
C11—N3—C10—N4	0.2 (4)	Zn2—N8—C32—C27	−177.0 (2)
C11—N3—C10—C9	180.0 (3)	C26—N8—C32—C31	177.4 (4)
C8—C9—C10—N4	35.1 (6)	Zn2—N8—C32—C31	0.9 (6)
C8—C9—C10—N3	−144.7 (4)	C28—C27—C32—N8	−179.4 (4)
C10—N3—C11—C12	−177.8 (4)	N7—C27—C32—N8	0.0 (4)
C10—N3—C11—C16	0.1 (4)	C28—C27—C32—C31	2.5 (6)
N3—C11—C12—C13	178.9 (4)	N7—C27—C32—C31	−178.1 (3)
C16—C11—C12—C13	1.3 (6)	C30—C31—C32—N8	−178.7 (4)
C11—C12—C13—C14	−0.3 (6)	C30—C31—C32—C27	−1.0 (6)

### Hydrogen-bond geometry (Å, °)

**Table:**

*D*—H···*A*	*D*—H	H···*A*	*D*···*A*	*D*—H···*A*
N2—H2···Cl3	0.86	2.64	3.316 (3)	137
N3—H3···Cl4^i^	0.86	2.76	3.291 (3)	121
N6—H6···Cl1^ii^	0.86	2.39	3.225 (3)	164
N7—H7···Cl2^iii^	0.86	2.52	3.277 (3)	148

Symmetry codes: (i) −*x*, *y*−1/2, −*z*+2; (ii) *x*, *y*, *z*+1; (iii) −*x*+1, *y*+1/2, −*z*+2.
